# Self-reported hepatitis C(HCV) testing among people living with human immunodeficiency virus (PLWH)

**DOI:** 10.1016/j.heliyon.2021.e07727

**Published:** 2021-08-06

**Authors:** Mustapha Thaim Buya Kamara, Veronica L Richards, Charurut Somboonwit, Haesuk Park, Nana Ayegua Hagan Seneadza, Zhou Zhi, Dushyantha Jayaweera, Emmanuel Thomas, Robert L Cook

**Affiliations:** a Department of Epidemiology, College of Public Health and Health Professions and College of Medicine, University of Florida, PO Box 100231, Gainesville, FL, 32610, USA; b Department of Internal Medicine, University of South Florida Morsani College of Medicine, Tampa, FL, USA; c University of Florida College of Pharmacy, Pharmaceutical Outcomes & Policy, Gainesville, FL, USA; d Department of Medicine, University of Miami Miller School of Medicine, 1501 NW 10th Avenue, Miami, Suit 811, FL 33136, USA; e Sylvester Cancer Center, University of Miami Miller School of Medicine, USA

**Keywords:** Infectious disease, Hepatitis, HCV, HIV/AIDS, Disparities, Testing

## Abstract

**Objective:**

We aimed to identify disparities in self-reported HCV testing among persons living with HIV (PLWH) in Florida.

**Methods:**

We utilized a cross-sectional study of 646 PLWH from the Florida Cohort study's baseline survey. Our analysis included chi-squared tests and logistic regression.

**Results:**

Participants that were 55 years old or above had more than twice the odds of reporting a past HCV test than those 18–34 years old (OR 2.47, 95% CI 1.22–5.0), which contrasted with Non-Hispanic Blacks who had lower odds of reporting a past HCV test than non-Hispanic Whites (OR 0.63, 95% CI 0.35–1.1). Drug use was also associated with higher odds of reporting a past HCV test for injection drugs (OR 2.9, 95% CI 1.0–8.43) and non-injection drugs (OR 1.52 CI 0.99–2.21). Individuals with education beyond high school had higher odds of reporting a past HCV test than those that did not attend/complete high school (OR 1.9 CI 1.11–3.16).

**Conclusion:**

Our findings highlight the success of the Center for Disease Control and the U.S. Preventive Services Task Force's campaign in groups at high risk of HCV, such as baby boomers and Injection Drug Users (IDUs). However, they also reflect the current low HCV testing in PLWH that are 18–34 years old, have a low level of education, and are non-Hispanic Black. Our findings are of crucial public health significance because untreated HCV in PLWH is a major cause of severe liver disease and death. They reveal the current deficiencies in HCV testing, which is the initial step to identify underlying reasons for inadequate testing in specific groups and develop practical solutions.

## 1 Introduction

In the United States, hepatitis C virus infection (HCV) affects approximately 0.93% of the general population [1]. However, HCV infection prevalence in people living with HIV (PLWH) can be as high as 25% [2]. PLWH infected with HCV have a higher risk of liver rigidity/cirrhosis than HIV-mono-infected patients. They are also more likely to progress to decompensated liver disease and death [3, 4]. Even with the higher prevalence of HCV infection and the risk of severe complications from co-infection, there is a current HCV testing gap in PLWH [5]. The Center for Disease Control (CDC) [6] and the United States Preventive Services Task Force (USPSTF) recommend one-time testing for HCV in PLWH [7], [p. 2]. Still, current testing levels are estimated to be 61%–82% [8, 9, 10, 11]. The testing gap of HCV is most evident in PLWH of specific sociodemographic categories, including minorities or individuals lacking higher levels of formal education [12, 13]. Minorities, including Hispanics and non-Hispanic blacks, have the highest HCV and HIV prevalence in the US [14, 15, 16] and are less likely to be referred and linked to HCV care after an HCV diagnosis. [12], [pp. 2009–2010] [17], A high prevalence and low HCV testing rates in these groups mean that they will not be diagnosed or be able to seek care early, leading to a rapid liver disease progression with subsequent cirrhosis and end-stage liver disease [18]. Additionally, the direct medical cost of chronic HCV infection was $10.7 billion between 2010 and 2019. The societal cost of premature mortality was $54.2 billion—the current average per-person Medicaid healthcare expenses for HCV infection range from $10,561 to $46,263. In PLWH, the cost is even more remarkable, placing them in the high-cost morbidity group.

HCV blood tests are essential in identifying persons exposed to the virus and are the initial step in the HCV care continuum, leading to a cure [19]. With proper education, persons exposed are empowered to seek adequate treatment and make healthy, informed behavioral decisions to prevent further disease progression and spread. However, due to the universal recommendation for HCV testing in PLWH by the Center for Disease Control (CDC) [6] and the United States Preventive Services Task Force (USPSTF) [20], healthcare professionals may do HCV tests without the patient's knowledge [21, 22]. For this reason, self-reports of past HCV tests in PLWH may differ from lab records. Using self-reported data instead of lab records, we focused on participants' awareness of their tests. The self-reported information enabled us to concentrate on factors that may influence participant's healthy habits, such as educational level and age. Consequently, this study aimed to utilize self-reported HCV testing history to identify testing disparities in a sample of PLWH in Florida, a state with one of the highest HIV prevalence [1].

## 2 Materials and methods

### 2.1 Population

The current study utilized a cross-sectional design consisting of PLWH, who participated in the Florida Cohort study's baseline survey. Details about the Florida Cohort include 932 PLWH recruited throughout Florida between October 2014 and December 2018. The sampling method included a non-probability convenience sampling with a prescreen added at the end of the recruitment to ensure a more representative sample of the population of PLWH in Florida [23]. Enrollment occurred in nine sites selected based on the investigators' geographic locations and recruitment centers' availability. The recruitment sites mainly included clinics, such as those run by the Florida Department of Health, a Federally Qualified Community Health Center, and academic settings. Recruitments also occurred in a food bank serving PLWH in South Florida. PLWH could self-refer in response to a brochure or be introduced to the team by a site's staff member. Research materials were in Spanish in two of the centers. Overall, Florida Cohort participants' age, race, and gender distribution were comparable to the overall population living with HIV in Florida. The only difference was no one was under 18 years and relatively few persons 60 years or older. Participants completed a questionnaire of demographics, health behaviors, substance use, and other variables linked to HIV health outcomes. All participants involved in the study provided written informed consent before enrollment. All participating Institutional Review Boards approved the study.

### 2.2 Sample size

The final sample consisted of 646 PLWH following the removal of 271 participants. Among those removed, 15 had no HCV data, 82 were unsure about having a previous test, and 174 had one or more missing values for any explanatory variable. We removed the uncertain participants because the interpretation of their responses could be misleading. There may be several unknown reasons they were unsure about their HCV test history, including fear of stigmatization or memory failure [24, 25, 26]. Participants selected answers to the questionnaire on paper or a computer. They could also write or type answers that were not listed.

### 2.3 Explanatory variables

These included factors that may be associated with testing for HCV, such as sociodemographic factors (age, sex, race/ethnicity, insurance status, education, employment, and marital status), alcohol use, non-injection drug use (including marijuana), injection drug use, and years since HIV diagnosis. [27], [pp. 2008–2016] [28].

### 2.4 Outcome

The primary outcome variable was past HCV test recorded from four responses to the question, "Have you ever been tested for Hepatitis C (HCV)?" The responses included "No," "Not Sure," "Yes-Result was positive," and "Yes-Result was negative," with the “Yes," denoting a past HCV test. We formed a new "Tested" group by combining the two “Yes” responses into one category to facilitate analysis. Therefore, our final groups for the outcome variable included the "Tested" and the "Not tested" categories. We did not ask about the type or number of past HCV tests in the questionnaire; therefore, participants may have indicated a screening or diagnostic test in their response. This distinction was irrelevant to our study.

### 2.5 Alcohol use

The AUDIT-C was used to assess for drinking status over the past 12 months [29]. We defined heavy drinking as consuming more than seven drinks per week (women) or more than 14 drinks per week (men). Moderate drinking included some level of drinking but not enough to reach the threshold of heavy drinking. We categorized no drinking as having no alcoholic drinks within 12 consecutive months.

### 2.6 Non-injection drug use

Non-injection drug users comprised of individuals who used marijuana and any other non-injection drug, including cocaine, crack cocaine, heroin (snorted or smoked), stimulants, pain medications, sedatives, and ecstasy or molly. We assessed marijuana by utilizing the question, "On average, how often have you used marijuana in the past three months?" Participants were considered users for the current study if they reported taking any marijuana during those three months. For other non-injection drugs, we utilized the question: "For each of the following non-injection drugs, please mark the response that best describes how often you used each drug in the past 12 months." We considered participants to have a history of taking non-injection drugs if they indicated that they had used them at least once in the past year.

### 2.7 Injection drug use

Injection drugs included heroin, cocaine, and stimulants. For this variable, we utilized the question, "Have you used drugs that you injected into your body with a needle that was not prescribed to you by a doctor?" We considered participants to have taken an injection drug in the past 12 months if they indicated they used it at least once in the past year.

## 3 Statistical analysis

We conducted descriptive analyses with the chi-square test as our preferred non-parametric test. Our sampling method explained above and the primarily nominal data with more than two levels made Fisher's test less desirable [30]. In addition, our binary outcome variable made multiple logistic regressions the most practical test to calculate the Odds Ratios and related confidence intervals. Based on a p-value cutoff of 0.10, variables selected from the chi-square test results for the multivariate logistic regression model included age, race, education, non-injection, and injection drug use [31]. Sex had a p-value that was more than 0.10 in the chi-square test results. However, we included it in the final multivariate logistic regression model due to the practical significance of sex differences in self-reported health-related data [32]. The final model included age (reference:18-34), sex (reference: male), race/ethnicity (reference: non-Hispanic, White), education (reference: less than high school), injection drug use (reference: no), and non-injection drug use (reference: no). There were missing data for non-injection drug use (n = 94), injection drug use (n = 40), and health insurance (n = 27). The missing data for non-injection drug users and injection drug users may not have been random, given the stigma associated with reporting illicit drug use. Participants may have skipped questions about drug use in the questionnaire due to the perceived fear of the consequences of drug use and to be more socially acceptable [33]. Nevertheless, utilizing complete-case analysis, we deleted all observations with missing values and examined results for bias [34, 35]. Estimates of parameters from our final sample showed minimal variation from those before the deletion, indicating negligible bias.

## 4 Results

The mean age of the sample was 46.4 years (SD = 11.1), and on average, participants had been living with HIV for 11.3 years (SD = 7.6). Eighty-three percent (537/646) of participants reported a past HCV test, and there were more males, 69% (446/646), in our sample. Both females and males had similar reports of past HCV tests, 82% (164/200) and 84% (373/446) respectively (p-value = 0.6085). Non-Hispanic Whites, 88% (131/149), and Hispanics, 87.5% (126/144) had statistically significant higher reports of past HCV tests than non-Hispanic Blacks, 79.4% (263/331) or others, 77.3% (17/22) (p-value = 0.0436). Eighty-four percent (216/257) of participants 45–54 years old and 89% (129/145) of participants 55 years old or above reported a past HCV test, which was higher than participants that were 18–34 years old, 77.9% (88/113) or 35–44 years old, 79.4% (104/131) (p-value = 0.0656).

More participants with health insurance reported a past HCV test 83.6% (510/610) than those without health insurance, 75% (27/36) p-value = 0.1803. IDUs (injection drug users) had higher reports of past HCV tests, 91.7% (44/48) than non-IDUs 85.2% (281/330) or non-drug users 79.1% (212/268) (p-value = 0.00378). Participants with education beyond high school had higher reports of past HCV tests, 86.4% (223/258), than those with only a high school diploma, 83.8% (155/185), or those that did not attend/complete high school, 78.3% (159/203) (p-value = 0.0670). Because of the weak and inconsistent bivariate associations and low practical significance to HCV testing of the remaining variables, including marital status, employment status, alcohol consumption, and the number of years living with HIV, there was no apparent need to further comment on their results (Table 1).

**Table 1:** Demographics and baseline characteristics of PLWH reporting past HCV test(s) (N = 646).

Frequency (%)	Frequency (%)		Unadjusted P-value^a^
	Not Tested for HCV (n = 109)	Tested for HCV (n = 537)	
**Age**			0.0656
18-34	25 (22)	88 (78)	
35-44	27 (20.6)	104 (79.4)	
45-54	41 (15.9)	216 (84)	
≥55	16 (11)	129 (89)	
**Sex**			0.6085
Male	73 (16.4)	373 (83.6)	
Female	36 (18)	164 (82)	
**Race/Ethnicity**			0.0436
Non-Hispanic, White	18 (12.1)	131 (87.9)	
Non-Hispanic, Black	68 (20.5)	263 (79.5)	
Hispanic	18 (12.5)	126 (87.5)	
Other	5 (22.7)	17 (77.3)	
**Insurance**			0.1803
Uninsured	9 (25)	27 (75)	
Insured	100 (16.4)	510 (83.6)	
**Education**			0.0670
Less than High School	44 (21.7)	159 (78.3)	
High School Diploma or Equivalent	30 (16.2)	155 (83.8)	
Beyond High school	35 (13.6)	223 (86.4)	
**Employment status**			0.8243
Unemployed	32 (17.8)	148 (82.2)	
Unable to Work/Disabled	47 (15.8)	249 (84.1)	
Employed	30 (17.6)	140 (82.3)	
**Marital Status**			0.3214
Married or Living with Long-Term Partner	25 (19.8)	101 (80.2)	
Single	84 (16.1)	436 (83.8)	
**Alcohol Use**^b^			0.7253
No drinking	32 (18.7)	139 (81.3)	
Moderate drinking	66 (16)	346 (84)	
Heavy drinking	11 (17.5)	52 (82.5)	
**Drug Use**^c^			
No Drug Use	56 (20.9)	212 (79.1)	0.00378
Non-Injection Drug	49 (14.8)	281 (85.2)	
Injection Drug Use	4 (8.3)	44 (91.7)	
**Years Since HIV Diagnosis**			0.2183
<10 years	59 (19.3)	246 (80.7)	
10–20 years	27 (13.4)	174 (86.6)	
>20 years	23 (16.4)	117 (83.6)	

a P-values come from chi-squared tests.

b Drinking status refers to past 12 months of drinking; we defined heavy drinking as >7 drinks/week (women) or >14 drinks/week (men); moderate drinking is any quantity below heavy drinking but >0 drinks over the past year.

c Use refers to the previous 12 months.

In the adjusted multivariate logistic regression analysis results shown in Table 2, we found that females and males had a negligible difference in their odds of reporting a past HCV test (OR 1.13, CI. 0.63–1.49). Participants 55 years old or above had more than twice the odds of reporting a past HCV test than those 18–34 years old (OR 2.47, 95% CI 1.22–5.0). Also, individuals with a history of injection drug use had higher odds of reporting a past HCV test than those without a history of drug use (OR 2.9, 95% CI 1.0–8.43). Likewise, non-IDUs had higher odds of reporting a past HCV test than non-drug users (OR 1.52, CI 0.99to 2.21). Also, participants with education beyond high school had higher odds of reporting a past HCV test than those that did not attend high school (OR 1.9, CI 1.11–3.16). In contrast, non-Hispanic Blacks had lower odds of reporting a past HCV test when compared to non-Hispanic whites (OR 0.63, 95% CI 0.35–1.12).

**Table 2:** Odds ratios for self-reported past HCV test(s) among persons living with HIV: multivariable logistic regression (N = 646).

	Odds Ratio^a^	95% Confidence Interval		p-values
**Age**				
18-34	Referent	Referent	Referent	
35-44	1.32	0.697	2.511	0.3923
45-54	1.687	0.941	3.026	0.0793
≥55	2.466	1.216	4.999	0.0123
**Sex**				
Male	Referent	Referent	Referent	
Female	1.127	0.705	1.801	0.6174
**Race/Ethnicity**				
Non-Hispanic, White	Referent	Referent	Referent	
Non-Hispanic, Black	0.628	0.353	1.118	0.1138
Hispanic	1.138	0.554	2.340	0.7250
Other	0.587	0.185	1.863	0.3658
Less than high school	Referent	Referent	Referent	
High School Diploma or Equivalent	1.48	0.872	2.511	0.4164
Beyond High school	1.87	1.106	3.161	0.0195
**Drug Use**^b^				
No Drug Use	Referent	Referent	Referent	
Non-Injection Drug Use	1.515	0.992	2.213	0.0542
Injection Drug Use	2.905	1.002	8.428	0.0497

a Values come from a logistic regression model, including age, sex, race/ethnicity, education, non-injection drug use, and injection drug use.

b Use refers to the previous 12 months.

## 5 Discussion

In our sample of PLWH, most of who have been living with HIV for over 11 years, only 83.1% reported getting an HCV test. This finding is consistent with previous studies testifying suboptimal HCV testing in PLWH [8, 11]. It also reveals the need to improve HCV testing in PLWH to ensure early detection of exposure and further management [6, 36]. More importantly, our findings showed testing disparities within various age groups, racial groups, educational levels, and drug use categories. Notably, participants 55 years and older and IDUs identified by the CDC and the USPSTF as being at high risk for HCV had greater odds of reporting a previous HCV test. This finding reveals the realization of the CDC's and USPSTF's HCV testing goals in PLWH. [37], [pp. 1945–1965] [38], However, specific subgroups of PLWH showed low HCV test rates. For example, Blacks who are more likely to be HIV-HCV co-infected [39] had low odds of reporting a past test which corresponds to previous studies on PLWH [12, 13, 15, 17].

The increase in HCV incidence caused by the opioid epidemic, which began in 2010, disproportionately affected young individuals. [40], [pp. 2006–2012] [41], A high incidence of HCV combined with low HCV testing that we discovered places exposed young people at increased risk for poor prognosis. Also, participants with incomplete or no high school education had lower odds of reporting a past HCV. This result also supports previous findings that a low level of education correlates to a low level of HCV awareness [12, 42]. These findings provide opportunities to target PLWH that are less likely to report a past HCV test and seek an understanding of the underlying cause(s). Whether a cause arises from the provider or the patient level, our results provide prospects to improve care in such a sensitive population. Adding user-friendly HCV testing notifications for PLWH in electronic medical records (EMR) may be one approach to improve care, particularly for those with reduced odds of reporting a previous HCV test. EMR has been utilized to enhance HCV care in the past for baby boomers, which resulted in the facilitation of sustainable awareness and improvement of HCV screening [6, 43]. In PLWH, improved screening and awareness comparable to that of baby boomers are also possible. Similarly, enabling better HCV-specific patient-provider communication through frequent training could promote HCV awareness in the lab, clinic, hospital, or other testing locations. Another strategy might be to create community engagement initiatives based on behavioral change theories used in clinics to enhance HCV education and self-management behaviors [44]. These theories are adapted to fit the objectives of community initiatives aimed at increasing HCV awareness. The goal would be to remove the hurdles to HCV testing and awareness.

Our study has numerous limitations, including a cross-sectional design, a small sample size, and data collection by self-report. Hence, we take our findings cautiously. However, since our sample included entirely PLWH, with the majority having viral suppression [23], we assumed recall bias would be minor. For example, participants are unlikely to claim that they were not tested for HCV because of their HIV diagnosis if they are stable [25]. Yet, they may report having an HCV test due to their HIV diagnosis, which may have led to an overestimation of the proportion of the tested group. On the other hand, it is conceivable that we underestimated the proportion of the tested group due to the failure of participants to recollect a previous test accurately or being unaware of one. Another limitation was the inability to include additional variables in our research. However, the variables included in the final analysis of the current study, such as age, sex, race, and education, are key factors that prevent access to HCV care [10]. Nevertheless, their role in those co-infected with HIV has had less attention [44].

Furthermore, we utilized unequal timelines for the sub-groups of non-injection drug users. We utilized the question from the survey that asked about marijuana use in the ‘last three months but included data for other non-injection drugs that asked about use over the ‘last year.’ Because we combined all non-injection drug use for analysis in this study, the timing difference may have understated the number of individuals who used marijuana outside the past three months. However, the proportion of marijuana users in our sample's preliminary analysis was similar to other studies [45]. Our final limitation was that we had a lower response rate for drug users since a reasonable number of participants did not answer the drug use questions. While we envisioned that this could cause biased estimates, further analysis showed that the loss was common in the tested than in the not tested group for both IDUs and non-IDUs. This pattern of missing values did not affect our findings for drug use since the odds of reporting a past test were higher in the tested group, albeit not significant.

Florida is one of nine states that constitute a cumulative HCV prevalence of 51.9% in the US, including California, Texas, New York, Pennsylvania, Ohio, Michigan, Tennessee, and North Carolina [1]. Therefore, our findings may not be generalizable to the entire United States. Still, they show deficiencies in testing or awareness in a region with a high HCV burden. Future research may include reviewing linked lab records in PLWH that could not report a past HCV test. Such a study could enable us to understand further reporting disclosures and any underlying reasons for the lack of reports in a population excluded from our research. It could also include exploring the underlying cause of the low self-reported HCV test in certain groups uncovered by the current study.

## 6 Conclusions

We discovered a suboptimal level of reported HCV testing in persons living with HIV for more than a decade. In addition, we identified low odds of reporting HCV tests in specific sociodemographic categories, including non-Hispanic Blacks, participants between 18 and 34, and those with incomplete or no high school education. These findings' public health significance includes providing opportunities for recognizing the underlying problem for low testing in specific groups and finding effective systems to solve them. Developing answers to such issues may help enhance HCV care in PLWH. They may also lead to lower healthcare costs from treating complications and mortality from liver disease.

## Declarations

### Author contribution statement

Mustapha Thaim Buya Kamara: Conceived and designed the experiments; Analyzed and interpreted the data; Wrote the paper.

Veronica L Richards, Nana Ayegua Hagan Seneadza and Zhou Zhi: Analyzed and interpreted the data.

Emmanuel Thomas, Dushyantha Jayaweera, Charurut Somboonwit and Haesuk Park: Conceived and designed the experiments.

Robert L Cook: Conceived and designed the experiments; Contributed reagents, materials, analysis tools or data.

### Funding statement

This work was supported by 10.13039/100000027National Institute on Alcohol Abuse and Alcoholism grants (U24 AA022002, U24AA022003, T32AA025877), and from the State of Florida via the 10.13039/100007698University of Florida (UF) and the 10.13039/100006686University of Miami Miller School of Medicine Institute for AIDS and Emerging Infectious Diseases.

### Data availability statement

Data will be made available on request.

### Declaration of interests statement

The authors declare no conflict of interest.

### Additional information

No additional information is available for this paper.
